# *Chlamydia pneumoniae* in Alzheimer's disease pathology

**DOI:** 10.3389/fnins.2024.1393293

**Published:** 2024-05-06

**Authors:** Lalita Subedi, Bhakta Prasad Gaire, Yosef Koronyo, Maya Koronyo-Hamaoui, Timothy R. Crother

**Affiliations:** ^1^Department of Pediatrics, Division of Infectious Diseases and Immunology, Guerin Children's at Cedars-Sinai Medical Center, Los Angeles, CA, United States; ^2^Infectious and Immunologic Diseases Research Center (IIDRC), Department of Biomedical Sciences, Cedars-Sinai Medical Center, Los Angeles, CA, United States; ^3^Department of Neurosurgery, Maxine Dunitz Neurosurgical Research Institute, Cedars-Sinai Medical Center, Los Angeles, CA, United States; ^4^Department of Biomedical Sciences, Cedars-Sinai Medical Center, Los Angeles, CA, United States; ^5^Department of Neurology, Cedars-Sinai Medical Center, Los Angeles, CA, United States

**Keywords:** Alzheimer's disease, amyloid beta-protein (Aβ) plaque, infection, microbes, *Chlamydia pneumoniae*

## Abstract

While recent advances in diagnostics and therapeutics offer promising new approaches for Alzheimer's disease (AD) diagnosis and treatment, there is still an unmet need for an effective remedy, suggesting new avenues of research are required. Besides many plausible etiologies for AD pathogenesis, mounting evidence supports a possible role for microbial infections. Various microbes have been identified in the postmortem brain tissues of human AD patients. Among bacterial pathogens in AD, *Chlamydia pneumoniae* (Cp) has been well characterized in human AD brains and is a leading candidate for an infectious involvement. However, no definitive studies have been performed proving or disproving Cp's role as a causative or accelerating agent in AD pathology and cognitive decline. In this review, we discuss recent updates for the role of Cp in human AD brains as well as experimental models of AD. Furthermore, based on the current literature, we have compiled a list of potential mechanistic pathways which may connect Cp with AD pathology.

## Introduction

Alzheimer's disease (AD) is the dominant cause of senile dementia, neuropathologically characterized by the presence of amyloid β-protein (Aβ) plaque and hyperphosphorylated (p)tau protein containing neurofibrillary tangles (NFTs), ultimately leading to neurodegeneration (Jack et al., 2018; Hampel et al., 2021; 2023 Alzheimer's disease facts figures, 2023). Late-onset AD (LOAD) is a progressive neurodegenerative disease that leads to the impairment of memory and cognitive ability, the erosion of social and behavioral skills, and eventual loss of life. The exact cause behind AD remains elusive but studies indicate that AD is ultimately the result of the sum of genetic, environmental, and lifestyle-related insults to the brain.

As AD mostly affects the aging population (Guzman-Martinez et al., 2019), per the National Institute of Aging (NIA), an estimated 6.7 million elderly Americans have been affected by AD dementia. AD is one of the leading causes of death in the elderly in the USA (2023 Alzheimer's disease facts figures, 2023). Worldwide, more than 50 million people have AD and trends suggest that these numbers may triple by 2050 (GBD 2019 Dementia Forecasting Collaborators, 2022). AD is more prevalent over the age of 65 with almost 50% of individuals aged over 85 being AD positive (2023 Alzheimer's disease facts figures, 2023). Significant progress has been made in diagnosis via FDA-approved PET-amyloid and -tau imaging, CSF Aβ, and more recently plasma phosphorylated tau 217 immunoassay (Ashton et al., 2024; Barthélemy et al., 2024) and disease-modifying treatments with anti-Aβ antibodies for AD, aimed at slowing down its progression (Dyck et al., 2022; Boxer and Sperling, 2023). However, despite these efforts, little headway has been achieved in finding both prophylactic and therapeutic AD treatments with substantial effects on preserving cognitive function.

The pathological hallmarks, Aβ plaques, NFTs, synaptic loss, neurodegeneration, vascular impairment, and more recently neuroinflammation, are all known to play a role in AD (Serrano-Pozo et al., 2011; Vickers et al., 2016). Aβ accumulation is believed to occur due to the hyperactivity of secretase enzymes (β-secretase and γ-secretase) which cleaves the amyloid precursor protein (APP) to Aβ40 and Aβ42 monomers. Oligomerization of these Aβ monomers results in the formation of amyloid plaque (Kravenska et al., 2020). Simultaneously, hyperphosphorylation of tau leads to the formation of NFTs in the limbic and cortical regions of the brain (Grundke-Iqbal et al., 1986). Subsequent accumulation of Aβ plaques and NFTs are associated with neurodegeneration affecting neurocognitive decline. Additionally, excessive accumulation of Aβ in the brain parenchyma attracts and activates glial cells, which subsequently potentiate neuroinflammation, vascular damage, and the infiltration of peripheral immune cells, further aggravating the neuroinflammation and neurodegeneration (Cai et al., 2014; Dionisio-Santos et al., 2019). Remarkably, the pathological hallmarks and subsequent inflammatory and neurodegenerative processes start many years before the onset of clinical symptoms (Bateman et al., 2012). This large preclinical gap provides an opportunity for early detection of disease processes, which is of paramount importance to allow effective early interventions before the irreversible loss of neurons.

The National Institute on Aging (NIA) and the Alzheimer's Association (NIA-AA) set a guideline for AD diagnosis and that is termed ATN; “A” stands for Aβ biomarker (amyloid PET or CSF Aβ42); “T” for the tau biomarker (CSF p-tau or tau PET); and “N” for the neurodegeneration biomarker (CSF t-tau, FDG-PET, or structural MRI) (Jack et al., 2016, 2018). Available detection methods limit the capacity to screen for early AD and predict progression and response to therapy in the clinical setting. Recent advances in the development and evaluation of brain PET imaging as well as plasma and cerebrospinal fluid (CSF) biomarkers have revolutionized the prospects of early diagnostic tools for AD (Li et al., 2015; Hameed et al., 2020). Both categories of fluid biomarkers appear superior to most currently available brain scanning tools, which require the administration of unsafe radiolabeled isotopes and have reduced cost-effectiveness, accessibility, and sensitivity. Yet, CSF extraction is an invasive approach which is not a convenient method for patients while plasma markers are affected by other peripheral metabolic processes, therefore plasma markers do not exclusively represent the events happening within the brain.

Recent evidence from experimental, epidemiologic, and clinical reports suggest that AD pathogenesis is not only restricted to the brain but also extends beyond the brain. The pathological hallmarks of AD are also identified in the retina, an accessible CNS tissue, in patients with mild cognitive impairment (MCI) and AD (Koronyo-Hamaoui et al., 2011; Morgia et al., 2016; Koronyo et al., 2017, 2023; Haan et al., 2018; Grimaldi et al., 2019; Shi et al., 2020, 2023; Xu et al., 2022; Walkiewicz et al., 2024). Beyond the CNS, AD pathogenesis is closely associated with several systemic abnormalities. Previous AD research generally focused on the CNS. However, several peripheral and systemic abnormalities, including disorders of systemic immunity, cardiovascular diseases, hepatic dysfunction, metabolic disorder, blood abnormalities, respiratory and sleep disorders, renal dysfunction, intestinal dysfunctions, and systemic inflammation are now understood to be linked to AD and are reported in AD patients (Wang et al., 2017).

### Microbial pathogens in AD

The exact cause of AD is unknown, yet several risk factors are thought to play synergistic roles in AD development. These factors include head injuries, age and sex, vascular disease, lifestyle, obesity/diabetes, environmental factors, genetic factors, and infection. With the recent increase in understanding of how inflammation and immune responses play a significant role in AD biology, infection has garnered increased attention in AD research since many of the pathological immune processes observed in AD are also driven by infections. One retrospective study found that severe infections, which are not just limited to the CNS but also systemic inflammation, increased the risk of vascular dementia and AD (Sipilä et al., 2021). This suggests that prophylactic and therapeutic treatment of infectious diseases might slow AD progression and cognitive decline in people with a risk of AD/dementia (Bu and Wang, 2021). Aβ itself is expressed in response to infections and can function as an anti-microbial peptide (Soscia et al., 2010; Kumar et al., 2016). This understanding led to the anti-microbial protection hypothesis of Alzheimer's disease which basically states that Aβ produced in response to infections, can over time become dysregulated leading to improper clearance and induction of inflammation and tangle formation (Moir et al., 2018). This pathway, over time, can then drive the development of AD. Therefore, it is enormously important to investigate the involvement of pathogens in AD progression to better understand AD pathogenesis and therapeutic intervention. Indeed, several studies have identified the presence of different microbes in the post-mortem brain tissue of AD patients, suggesting they might be involved in AD (Renvoize and Hambling, 1984; Miklossy, 1993, 2011, 2015; Itzhaki et al., 1997; Riviere et al., 2002; Alonso et al., 2014; Pisa et al., 2015; Perry et al., 2016; Dominy et al., 2019; Ciaccio et al., 2021; Senejani et al., 2021).

Dr. Oscar Fischer was the first to describe in 1907 that cerebral plaques may be the result of a chronic infection and suggested the infectious etiology of AD (Fisher, 1907; Broxmeyer, 2017). Among the pioneer studies that provided evidence that infections playing a role in AD were conducted by Itzhaki et al. (1997), observing herpes simplex virus 1 (HSV1) in the AD brain and revealing HSV1 as a risk factor for AD. A few other reports also support the presence of HSV1 in AD brains (Chiara et al., 2012; Itzhaki, 2014). Another study observed an association with the presence of serum antibody titers for cytomegalovirus in AD patients (Renvoize and Hambling, 1984). More recently, many studies have suggested that COVID-19 may play a role in the onset of dementia and AD (Ciaccio et al., 2021; Pyne and Brickman, 2021; Gordon et al., 2022). SARS-CoV-2 virus infection can activate AD-like signaling in the human brain and induce neuroinflammation (Reiken et al., 2022; Soung et al., 2022). In addition to viruses, bacteria that can reach the brain generally will induce neuroinflammation, and subsequent neurodegeneration which are the major cascades of AD pathology (Balin et al., 1998; Lim et al., 2014; Tran et al., 2022). A series of investigations found different kinds of spirochetes in human AD brain tissues, possibly revealing a strong connection of these microbes with AD pathogenesis as well (Miklossy, 1993, 2011, 2015). *Borrelia burgdorferi*, the causative agent of Lyme disease, was detected in AD brains, as a co-localized form with amyloid markers (MacDonald and Miranda, 1987; Senejani et al., 2021). *Porphyromonas gingivalis*, a pathogen for chronic periodontitis, was also observed in the brain tissues of AD patients (Dominy et al., 2019). Different oral Treponema species have been observed in the trigeminal ganglia, pons, and hippocampus (Riviere et al., 2002). *Helicobacter pylori* (Hp) IgG titer was higher in AD patients compared with control patients (Malaguarnera et al., 2004), and epidemiological studies suggested that it is involved in the activation of innate immunity pathways that can exacerbate the CNS system leading to AD-like complications (Park and Tsunoda, 2022). Other studies also show a positive relation between Hp infection and AD (Park et al., 2017; Liu et al., 2021). *Toxoplasma gondii*, a eukaryotic pathogen, has been suggested to be involved in AD (Kusbeci et al., 2011; Mahami-Oskouei et al., 2016; Perry et al., 2016). Other microbes that were detected in the AD brains are Human herpes virus-6, Varicella zoster virus, HCV, and Influenza virus. Additionally, various fungal (e.g., *Candida glabrata, Candida famata, Syncephalastrum racemosum, and Candida albicans*) pathogens have also been observed in AD brains (Alonso et al., 2014; Pisa et al., 2015). Overall, viruses, bacteria, and fungi, as well as single-cell eukaryotes have been detected in human AD brains suggesting that AD is possibly manifested by multiple microbial infections in the body/brain. Highlighting this, one study found that vaccination for tetanus, diphtheria, and pertussis reduced the risk of dementia in adults (Scherrer et al., 2021). Furthermore, there is ample evidence that the BCG vaccine may provide a protective effect on AD development (Zuo et al., 2017; Gofrit et al., 2019; Greenblatt and Lathe, 2024).

### Cp infection and AD pathogenesis

**Figure 1:**
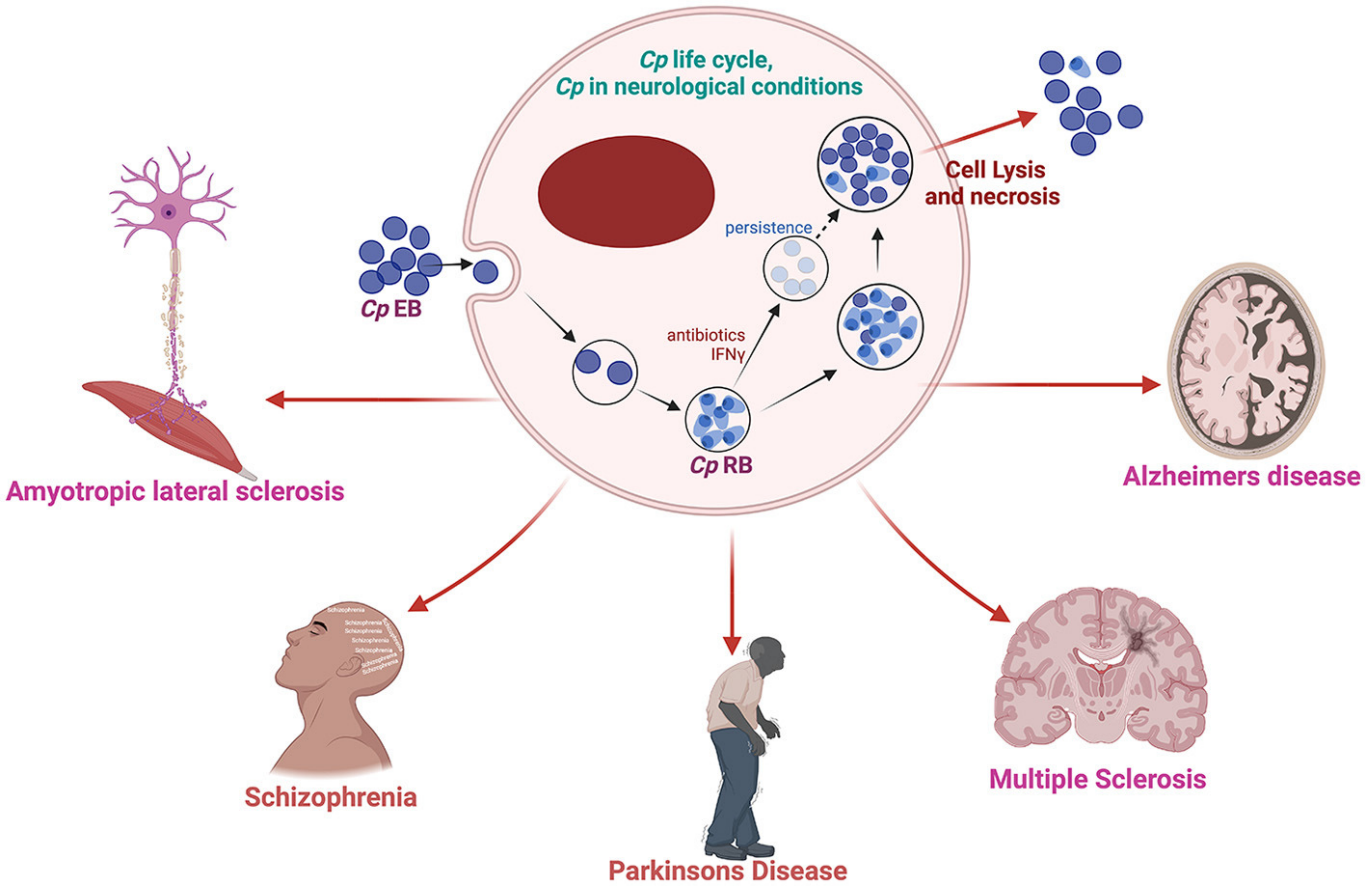
Schematic diagram of Cp life cycle and its possible involvement in several neurological complications. Created with BioRender.com.

Among the bacterial infections detected in the brain of AD patients, Cp is the most consistent and promising candidate for involvement in AD (Balin et al., 1998; Paradowski et al., 2007). Cp is a gram-negative obligate intracellular bacterium that causes upper and lower respiratory tract infection and is a major contributor to community-acquired pneumonia (Miyashita et al., 2001). Cp has a unique lifecycle which includes a metabolically inactive elementary body that enters the cell forming a compartment termed an inclusion. The inactive elementary body then changes to the metabolically active reticulate body which then replicates, eventually changing back to the elementary body which is released from the cell-by-cell lysis. Chlamydiae all possess type III secretion systems which are required for host cell manipulation and immune system evasion (Peters et al., 2007). Under immune or antibiotic pressure, Cp can transform into a more persistent state, hiding inside the cell until conditions improve (Malinverni et al., 1995). However, little research has been performed in this area. Cp has been suggested to play a key role in several aging-related conditions including AD (Figure 1) (Kuo et al., 1995; Porritt and Crother, 2016).

Brian Balin and Alan Hudson first observed Cp in AD brains (Balin et al., 1998) and similarly, other studies also reported the presence and increased expression of Cp-associated markers in AD brains correlating with neuropathology, suggesting a causal link (Chiara et al., 2012; Balin and Hudson, 2014). Cp was observed in close association with both amyloid plaques as well as NFTs (Balin et al., 1998; Gérard et al., 2006; Hammond et al., 2010). Immunohistological studies placed Cp within neurons, microglia, astrocytes, and pericytes (Balin et al., 1998; Gérard et al., 2006; Hammond et al., 2010). In some cases, live Cp was grown from AD patient brain tissue samples by infecting Hep2 cells using the postmortem AD brain tissue lysate (Balin et al., 1998; Gérard et al., 2006; Dreses-Werringloer et al., 2009). An ultrastructural analysis of Cp in AD brains determined that the Cp did not appear as classical inclusions but may have been in a more persistent state (Arking et al., 1999). Additionally, various studies have observed increased anti-Cp titers in AD patients compared with normal controls (Yamamoto et al., 2005; Bu et al., 2015). Meta-analyses of the various studies seeking to determine relationships between infectious organisms and AD have placed Cp as the likeliest organism to play a role in AD pathogenesis (Maheshwari and Eslick, 2015; Ou et al., 2020). As depicted in Table 1, a growing body of studies found Cp as a potential contributor to AD pathogenesis.

**Table 1:** Cp in AD: human studies.

**SN**	**Sample size**	**Cp+**	**Detection method**	**Conclusion: Cp infection**	**References**
1	AD: 19 NC: 19	AD: 19 NC: 1	PCR, IHC, IEM, Culture	Cp detected in all AD brains	Balin et al., 1998
2	AD: 21	AD: 21	PCR, Brain	APOE4 > non APOE4 AD patients	Gérard et al., 2005
3	VAD: 31 AD: 61 NC: 32	AD: 22 NC: 8	ELISA, serum	Cp-CRP in AD	Yamamoto et al., 2005
4	AD: 25 NC: 27	AD: 20 NC: 3	PCR, Brain	Majority of AD brains	Gérard et al., 2006
5	AD: 57 VD: 21 NC: 47	AD: 25 NC: 5	PCR, CSF	Cp, tau, and Abeta42 in CSF	Paradowski et al., 2007
6	AD:2	AD: 2	PCR, culture	Respiratory type Cp in AD	Dreses-Werringloer et al., 2009
7	AD: 5 NC: 5	AD: 5 NC: 2	IHC and Thioflavin S staining	Cp antigens in cortices, Cp*-associated* Aβ and NFT in AD	Hammond et al., 2010
8	AD: 128 NC: 135	AD: 112 NC: 98	ELISA, Serum	High Aβ, IFN-γ, TNF-α, IL-1β IL-6, etc.	Bu et al., 2015
9	Cp-infected patients: 1,657, control: 4971	95% of Cp-infected people: high AD risk	Nationwide cohort study	Cp-pneumonia adds high AD risk	Ou et al., 2021
10	AD:339 NC: 339	AD: 209 NC: 205	ELISA, Serum	Cp-Seropositivity in AD patients	Lindman et al., 2021
11	AD: 17 NC: 9412	NA	Median fluorescence intensity (MFI) in serum	Serum *C. trachomatis* (CT) antigens were higher in AD patients	Lehrer and Rheinstein, 2022
12	AD: 21 NC: 10	AD: 18 NC: 0	PCR, IHC staining of Brain	Cp in AD brain was linked with Tau-related NFT	Mahony et al., 2000
13	AD: 82	AD: 5	(IFA, Serum)	Doxycycline and rifampin treatment improved AD conditions	Loeb et al., 2004
14	AD: 20	AD: 0	PCR, IHC	Unable to detect Cp in AD brains	Gieffers et al., 2000
15	AD: 4 NC: 16	AD: 0 NC: 0	PCR, brain	Unable to detect Cp in AD brains	Wozniak et al., 2003
16	AD: 12 NC: 13	AD: 0 NC: 0	PCR, IHC, brain	Unable to detect Cp in AD brains	Nochlin et al., 1999
17	AD: 15 NC: 5	AD: 0 NC: 0	PCR, brain	Unable to detect Cp in AD brains	Ring and Lyons, 2000
18	AD: 9 NC: 2	AD: 0 NC: 0	PCR, IHC brain	Unable to correlate Cp with LOAD.	Taylor et al., 2002

While many studies observed the presence of Cp in human AD brains, some studies failed to identify its presence (Nochlin et al., 1999; Gieffers et al., 2000; Ring and Lyons, 2000; Taylor et al., 2002; Wozniak et al., 2003). However, this is understandable as even if Cp does play a role in AD pathogenesis, it is unlikely to be a universal mechanism, as other infections, environmental, and genetic factors will play important roles. Additionally, differences in methodologies could also explain the lack of Cp detection. For example, Paradowski et al. analyzed the level of soluble Aβ in AD patients with Cp infection but were not able to find any difference between AD patients that were Cp-positive and Cp-negative (Paradowski et al., 2007). Several factors including sample location bias, amount of DNA loaded, sample size, etc., might contribute to variation in the results. Overall, there are several limitations in the methods utilized (culture, PCR, IHC, EM etc.) to interrogate the presence of Cp in the human brain, including location bias relative to the large sample size. Some studies used fresh tissue and other used paraffin-embedded tissue. Paraffin embedded tissues, which are generally more common, provide a more difficult route for experimental detection of Cp. Nonetheless, the majority of studies did find a correlation between Cp infection and Aβ oligomers in AD patients (Table 1).

Besides human studies, several *in vivo* (Table 2) AD mouse model studies obtained evidence for a role of Cp in AD pathogenesis. Most of the *in vivo* experimental studies employ intranasal administration of Cp to investigate the role of Cp infection in AD-related pathologies in mice. Intranasal Cp infection in mice leads to increased Cp invasion of the brain and accelerated Aβ accumulation in the brain (Little et al., 2004, 2005, 2014; Boelen et al., 2007a; Voorend et al., 2010; Chacko et al., 2022). In addition, a few studies suggested neuronal accumulation of Aβ following Cp infection and glial cells as the host for Cp in the CNS (Little et al., 2004; Chacko et al., 2022). These studies from AD animal models found that intranasal Cp infection can reach different parts of the brain inducing increased amyloid deposition, glial activation, and triggered neuroinflammatory cascades which all could lead to the aggravation of AD pathogenesis. However, to date, no actual cognitive studies have been performed using an AD transgenic model with Cp infection.

**Table 2:** Cp in AD: mouse studies.

**SN**	**Mice (age, sex, and strain)**	**Infection (bacteria, route, and dose)**	**Observation**	**Bacterial strain**	**References**
1	3-month, female BALB/c	Cp, IN, 2–4 × 10^4^ IFU	↑ brain Aβ, ↑ intracellular Aβ1–42 in neuron	96–41 isolate from human AD brain	Little et al., 2004
2	3-month, female, BALB/c	Cp, IN, 10^7^ IFU	↑ Cp in OB and brain, ↑ brain Aβ aggregates.	TWAR2043	Boelen et al., 2007a
3	8-week, female BALB/cJ	Cp, IN, 5 × 10^5^ IFU	Cp and Aβ deposition Viable Cp cultured from OB 4-month pi	AR39	Little et al., 2014
4	7–8-week, female BALB/c	Cp, IN epithelium injury model, 1 × 10^6^ IFU	Cp in OB and brain within 24 h of infection Cp reside in glial cells as their host Cp associated with ↑ Aβ aggregate. ↑ Cp in brain-altered AD-related genes	AR39	Chacko et al., 2022
5	Within 24 h of birth, Males and females, BALB/c	*Chlamydia muridarum*, IN, 400 IFU	Male mice hippocampus showed ↑ brain Aβ CRH and ↓ in vasopressin, hypocretin, and oxytocin Female mice hippocampus showed ↑ in prolactin (7.51-fold), oxytocin (4.92-fold), hypocretin (9.51-fold), and vasopressin (13.07-fold)	VR-123	Wynne et al., 2011
6	6- and 20-month-old, Female, BALB/c	Cp, IN, 5 × 10^4^ IFU	Cp in heart, brain, and OB Cp-infection load were high in aged mice	AR-39	Little et al., 2005
7	3-month, ApoE KO, ApoE/LDLr KO, and C57BL/6J	Cp, IP, 3 × 10^7^ IFU.	Cp DNA in brain	TWAR 2043	Voorend et al., 2010

↑ increase, ↓ decrease.

In addition to mouse models and post-mortem human tissue samples, many *in vitro* studies have been performed which support a relationship between Cp and AD (Table 3). Cp infection may lead to increased BBB permeability and inflammatory responses of immune cells to trigger disease pathologies, thereby exacerbating AD. For instance, Cp infection to Human brain microvascular endothelial cells (HBMECs) increased the level of N-cadherin, VE-cadherin, β-catenin, VCAM, and ICAM (MacIntyre et al., 2002, 2003). Monocytes infected by Cp *in vitro* increased their migration and amounts of adhesive molecules like LFA-1, VLA-4, and MAC-1 (MacIntyre et al., 2003). These molecules are associated with the infiltration of peripheral immune cells into the brain. Additionally, Cp infection of THP1 cells led to increased production of IL-1β, IL-6, and IL-8, which have been closely associated with sporadic/LOAD) (Lim et al., 2014). Human astrocytoma infected with Cp led to the activation of the pro-amyloidogenic pathway by enhancing APP processing, increased the activity of β-secretase and γ-secretase, and reduced α-secretase, which eventually increased Aβ production by cleaving APP (Al-Atrache et al., 2019).

**Table 3:** Cp in AD: *in vitro* studies.

**SN**	**Cell type**	**Cp dose and strain**	**Observation**	**References**
1	Human brain microvascular endothelial cells (HBMECs)	AR-39, 5 × 10^5^ IFU	↑ N-cadherin, VE-cadherin, and β-catenin	MacIntyre et al., 2002
2	HBMECs and human monocytes (THP-1)	AR-39, MOI ~ 0.05–0.2	↑ Monocyte migration, ↑ VCAM-1 and ICAM-1 on HBMECs and ↑ in LFA-1, VLA-4 and MAC-1 on monocyte	MacIntyre et al., 2003
3	Neuroblastoma (SK-N-MC)	AR39, MOI 1	Chronic infection and ↓ neuronal apoptosis	Appelt et al., 2008
4	THP1	AR39, MOI = 1	Innate and adaptive immune response IL-1β, IL-6, IL-8 ↑Inflammation related to sporadic/LOAD	Lim et al., 2014
5	Human astrocytoma (CCF-STTG1)	AR39, MOI = 1	↑ Pro-amyloidogenic pathway of APP processing, ↑β-secretase, ↑γ-secretase, and ↓α-secretase	Al-Atrache et al., 2019
6	Computational modeling on the olfactory system	NA	Cp can move from the olfactory tract to the olfactory cortex and the hippocampus and cause AD	Sundar et al., 2020
7	SH-SY5Y, HEp-2, HMC3, THP-1	AR-39, MOI: 1	↑ IL-1β, Il-8, TNF- α ↑ Microglia activation ↑ Neuroinflammation	Kaya-Tilki and Dikmen, 2021
8	Primary mouse OECs, TgSCs, astrocytes, and microglia	AR39, MOI:1	Infect, survive, and replicate within glia (astrocytes and microglia) from the PNS and the CNS Increase Aβ deposition	Chacko et al., 2022
9	Human astrocytoma and microglioma cell lines U-87 MG and CHME-5 (respectively)	AR-39	Astrocytes and microglial infection with Cp look like that of Hep-2 Cp displays an active phenotype rather than the persistent form in both cells	Dreses-Werringloer et al., 2006
10	Astrocyte, neuron, and microglia	TWAR 2043, MOI: 5	Neurons are more sensitive to Cp and ↑ necrosis and death Astrocytes ↑ extracellular Cp and ↓ necrosis Microglia are highly resistant to Cp and act as persistent hosts	Boelen et al., 2007c
11	Murine microglial cell (BV2), murine astrocyte cell, murine neuroblast	TWAR 2043, MOI: 5	Cp antigen found in infected microglia and astrocytes Cp infection ↑ level of MCP1, IL-6, TNF-α IL-1β in microglia Cp-infected microglia induced apoptosis and necrosis to neuronal cells	Boelen et al., 2007b
12	Microglial cell (EOC 20)	TW 183, MOI: 10	↑ TNF- α and MMP-9 but not MMP-2	Ikejima et al., 2006

↑ increase, ↓ decrease.

### Possible mechanisms by which Cp infection affects the brain

There are likely three major routes by which Cp can affect brain biology. (1) The first route is via systemic interactions. Acute infection of the lung can lead to systemic cytokine production, which in the context of the right inflammatory signals, can pass into the brain directly. Additionally, elementary bodies released from infected cells can pass into the circulatory system leading to infections in other tissues and organs. (2) The second route for Cp migration is via a Trojan horse. As an obligate intracellular pathogen, Cp resides in cells, including immune cells such as monocytes/macrophages, T-cells, and neutrophils, and infected cells can migrate to other parts of the body, including the brain, thereby spreading the infection (Kortesoja et al., 2020). Cp*-*infected neutrophil granulocytes can also be taken up by macrophages silently helping Cp for its replication and hindrance against the immune challenge from the human host (Rupp et al., 2009). These macrophages can migrate to the brain, eventually releasing Cp there. Additionally, while infiltrating macrophages/monocytes can either exacerbate disease via inflammatory pathways, or aid in Aβ clearance (Butovsky et al., 2006; Koronyo-Hamaoui et al., 2009, 2019; Koronyo et al., 2015; Li et al., 2020), infected macrophages are likely to only promote disease pathology. (3) The third route is a direct infection of the nasal cavity. Cp has been shown to directly infect the brains of mice via the nasal route and damage to these areas can lead to enhanced delivery of Cp into the brain (Chacko et al., 2022). The trigeminal nerve extends between the olfactory bulb and the brain and acts as an invasion path through which Cp can readily pass and reach the CNS (Chacko et al., 2022). Cp that reaches the brain can infect the brain's resident cells and trigger proinflammatory signals through activated glial cells (microglia and astrocytes) resulting in secondary brain damage by inducing neuroinflammation. None of these three mechanisms are mutually exclusive and likely all play some role to varying degrees.

## Role of Cp in resident brain cells

Under homeostatic conditions resident brain cells work in concert to maintain the optimal CNS environment. However, under neurological disease conditions, such as AD, brain-resident glial cells are activated resulting in a neuroinflammatory milieu, which can subsequently lead to neurodegeneration. Previous studies suggest that Cp can infect both glial and neuronal cells in the brain. Cp can infect and survive in cultured mouse primary microglia, astrocytes, and OECs (Chacko et al., 2022). Furthermore, Cp could also replicate in peripheral (OECs and TgSCs) and well as CNS glial cell types from the peripheral nervous system and the CNS (astrocytes and microglia) (Chacko et al., 2022). Similarly, Cp can infect and reproduce in human microglia and astrocytes *in vitro*, as well as neuronal cells (Dreses-Werringloer et al., 2006; Boelen et al., 2007c). Neuronal cells were the most permissive to Cp infection, while microglial cells were less but still contained Cp DNA, suggesting these cells may induce a more persistent Cp phenotype (Boelen et al., 2007c). Here, we discuss in greater detail the potential role of Cp in these individual CNS cell types.

### Glia (microglia and astrocytes)

**Figure 2:**
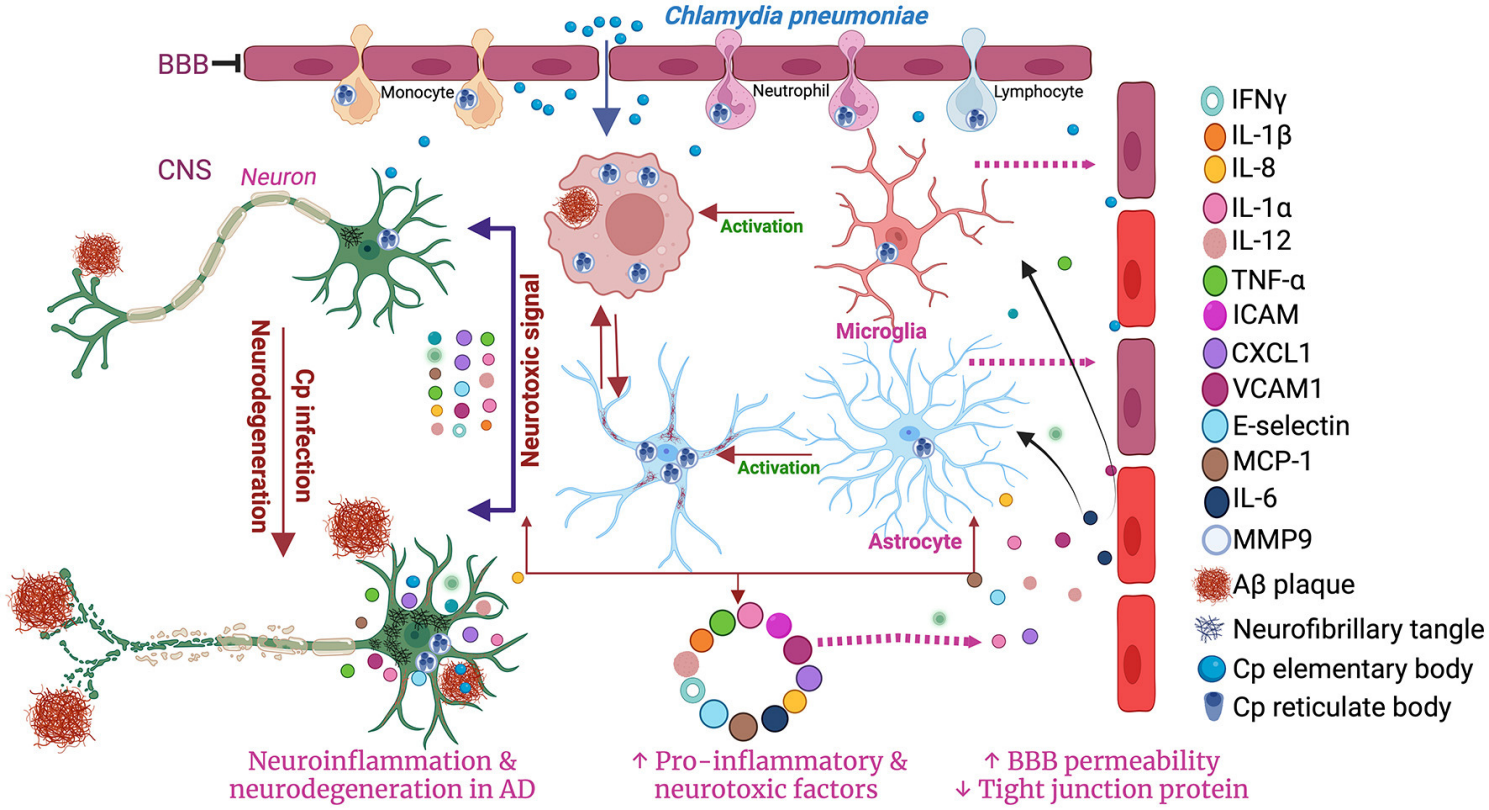
Schematic diagram of the involvement of microglia and astrocytes in Cp-mediated neuroinflammation. Created with BioRender.com.

A study performed in post-mortem human brain observed the presence of Cp antigens inside both microglia and astrocytes (Balin et al., 1998; Arking et al., 1999; Gérard et al., 2006; Hammond et al., 2010). As an immune cell and a professional macrophage, microglial cells are known to be more resistant to infection, including Cp infection. However, Cp DNA has been found in microglia exposed to Cp (Boelen et al., 2007c). Cp infection in murine microglia and astrocytes both led to an increase in the level of several inflammatory cytokines and chemokines in the culture medium. Cp-infected microglia released a greater amount of TNF-α and IL-1β while Cp-infected astrocytes released a greater amount of monocyte chemoattractant protein 1 (MCP-1), and IL-6 compared with controls (Boelen et al., 2007c). Treatment of neuronal cells with Cp-infected microglial and astrocyte-conditioned media resulted in neurotoxicity *in vitro* (Boelen et al., 2007b). Microglial supernatant was found to be more neurotoxic than astrocyte supernatant following Cp infection, indicating that microglia can produce neurotoxic factors following Cp infection (Boelen et al., 2007b). Similarly, Cp infection of EOC microglial cells also led to the production of proinflammatory cytokines like TNF-alpha and selectively induced matrix metalloproteinase-9 (MMP9) (Ikejima et al., 2006). Cp-induced microglial activation in the hippocampus was observed in a mouse model of atherosclerosis where Cp was injected i.p. (Voorend et al., 2010). These data support the idea that in addition to the direct nasal route, Cp can access the brain via the BBB likely residing in trafficking immune cells. Previous reports suggested that many toll-like receptors, including TLR1, 2 4, and 6 are expressed in microglia (Laflamme and Rivest, 2001; Laflamme et al., 2001; Bsibsi et al., 2002). These receptors are reported to be involved in the Cp-mediated inflammatory cascades in a MYD88-dependent manner (Porritt and Crother, 2016). Cp elementary bodies can activate these receptors to accelerate the downstream inflammatory pathways through MYD88 resulting in the production of many inflammatory mediators and cytokines including IFN-γ, IL-1α, IL-18, IL-1β, TNF-α, IL-6, IL-12, MCP-1, CXCL1, ICAM1, VCAM-1, E-Selectin, etc. (Porritt and Crother, 2016). These findings suggest that microglia infected with Cp can drive inflammatory responses in the brain (Figure 2).

While less is known about Cp and astrocytes, *in vitro* studies have found that Cp grow better in astrocytes than in microglia (Boelen et al., 2007b). Astrocytes provide lipids for neurons myelin sheaths which may make these cells more hospitable for Cp growth. Cp-infected astrocytes have altered expression and activity of secretases which are critical to β-amyloid generation in AD (Al-Atrache et al., 2019). Cp infection induced the pro-amyloidogenic pathway of APP processing via increasing the expression and activity of β-secretase and γ-secretase and decreasing the expression and activity of α-secretase. These effects of Cp infection in astrocytes provide evidence for a direct link between Cp and AD pathology (Al-Atrache et al., 2019). Therefore, further studies need to be performed investigating the relationships between Cp infection and astrocytes.

### Neurons

Contini et al. suggested that neuronal cells act as the host for the Cp in AD brains and infected neuronal cells were observed to be near NFT and NSP in AD brains (Contini et al., 2010; Balin et al., 2018). In one study, intracellular Cp was observed in almost 1% of the brain cells including neuroglia, different kinds of neurons, and peri-vascular cells (Hammond et al., 2010). Though the presence of Cp was observed in the neuron of humans as well as an animal model of AD its role in the neuron has yet to be explored. In *in vitro* studies, neuronal cells were most permissive to Cp infection. As mentioned above, supernatants from Cp-infected microglia were found to be neurotoxic (Boelen et al., 2007b). A few studies, however, reported that direct Cp infection can lead to the inhibition of apoptosis in neuronal cells (Appelt et al., 2008). Nonetheless, Cp-infected immune cells can promote neurodegeneration by increasing the production of inflammatory mediators (Boelen et al., 2007b).

### Oligodendrocytes and endothelial cells

Unfortunately, no studies to date have been performed investigating Cp infection and oligodendrocytes. However, it is likely that these cells are permissive to some degree for Cp infection. While there is no data regarding Cp and brain endothelial cells, there is large a set of data which support an association between Cp infection and atherosclerosis (Grayston et al., 1995; Belland et al., 2004; Campbell and Kuo, 2004). Growing evidence suggests that in endothelial cells Cp can induce oxidative stress in mitochondria, downregulate MMP3/MMP9 synthesis, and increase TLR2 signaling (Pietro et al., 2013; Wang et al., 2013; Ma et al., 2015; Zhao et al., 2022). These biological events promote the migration of vascular smooth muscle cells to promote atherosclerotic plaque. Cp infection in human brain microvascular endothelial cells induced the expression of adhesion-related proteins, zonula adherent proteins, and reduction of blood-brain barrier tight junction proteins including beta-catenin, N-cadherin, and VE-cadherin, suggesting that Cp can induce the permeability of endothelial cells and possibly leads to BBB permeability (MacIntyre et al., 2002).

### *Chlamydia pneumoniae* in AD: causative or accelerating agent?

The role Cp may play in AD pathogenesis is still a topic of discussion and requires further studies. Among the studies that observed Cp in the AD brain, most found Cp in the brain of late-stage AD patients. Based on emerging research findings, scientists suggest that a persistent form of Cp might play an important role in the induction and progression of AD by increasing the formation and accumulation of senile plaque and NFTs and by increasing neuroinflammation (Balin et al., 1998). Indeed, some studies revealed that Cp can increase the production of Aβ both in animal models and *in vitro* cultures (Little et al., 2004; Boelen et al., 2007a; Al-Atrache et al., 2019; Chacko et al., 2022). This increase in amyloid production in response to infection supports the antimicrobial protection hypothesis of AD (Soscia et al., 2010; Kagan et al., 2012; Kumar et al., 2016; Moir et al., 2018) Aβ has been found to play a role as an antimicrobial peptide acting as a trap for microbes. However, the multiplication of microbes and the availability of the immune system to defend against these conditions and to drain the complex of Aβ with trapped microbes are limited. Rather, such a condition may lead to the accumulation of Aβ and activation of inflammatory cells leading to aggravated neuroinflammation. This neuroinflammation could cause secondary damage to neurons and other immune cells present in the CNS besides the infection itself. Further, as mentioned above, conditioned media from Cp-infected microglia cell lines possessed neurotoxicity (Boelen et al., 2007b).

As mentioned before, one likely route for Cp to gain access to the brain is through the nasal cavity (Chacko et al., 2022). Once there, it can increase AD-like pathology in the brain characterized by increased Aβ, Tau, and neurodegeneration (Boelen et al., 2007a). Intranasal infection with Cp resulted in the formation of amyloid-like deposits in the brain of the non-AD mouse models. However, Cp infection in the AD brain was correlated with lower CSF AB42 levels and higher CSF tau protein suggesting that the presence of Cp might differentially regulate the Aβ42 and tau in AD brains (Paradowski et al., 2007). Aβ1–42 immunoreactive deposits were observed in brains of Cp-infected mice. The extent of infection was proportionate to the number, size, and density of Aβ1–42 deposits in the infected brains (Little et al., 2004). These findings reaffirm that Cp-mediated infection may be involved in the pathogenesis of sporadic AD. Altogether, these data indicate that Cp can reach and infect cells in the brains of both mice and humans and provide evidence to support the hypothesis that Cp can play a role in AD pathogenesis. This is likely mediated directly by neuronal cell infection and cell death and indirectly by overactivation of glial cells and their responsive inflammatory cascade-mediated neuroinflammation. All these events are capable enough to affect the hippocampus as well as to induce AD-like pathology in the long run. Additionally, this would also open the possibility that CP can initiate as well as aggravate AD.

## Potential mechanistic pathways where Cp can affect AD development and progression

### APP/Aβ

The presence of Aβ was the first major discovery into the mechanisms of AD pathogenesis. Amyloid precursor protein (APP) is responsible for the production of neurotoxic Aβ via proteolysis which results in AD pathology (O'Brien and Wong, 2011). Accumulation of Ab leads to plaque formation, which is believed to initiate the conversion of tau from the normal to toxic form, which mediates the Aβ toxicity at the synapse (Bloom, 2014). Excessive APP accumulation in/near mitochondrial import channels results in mitochondrial dysfunction, which is a key event of AD pathology (Devi et al., 2006). In contrast, Aβ peptide possesses antimicrobial properties as well and is expressed in response to infections, a key component of the anti-microbial hypothesis of AD (Soscia et al., 2010; Kagan et al., 2012; Kumar et al., 2016; Moir et al., 2018) Cp infection in human astrocytes dramatically increases APP suggesting that Cp infection can trigger APP production and subsequent AD pathology (Al-Atrache et al., 2019). Cp infection can induce Aβ in the brains of mice (Little et al., 2004; Chacko et al., 2022). Several studies identified the presence of Cp in the brain tissue of AD patients and induce the increased production of Aβ plaque and AD-like conditions (Yamamoto et al., 2005; Gérard et al., 2006; Paradowski et al., 2007; Chacko et al., 2022). It is unknown if Aβ plays any role controlling Cp infection, but it is likely that Cp could influence AD development by inducing excessive Ab production.

### APOE4

The apolipoprotein E (APOE) ε4 allele (APOE4) is a leading risk determinant for AD with a host of detrimental interactions (Yamazaki et al., 2019; Raulin et al., 2022), the APOE4 derived from astrocytes are reported to be responsible for the alteration of BBB integrity during AD pathology (Jackson et al., 2021). APOE4 has been associated with an increased burden of Cp in human AD brains (Gérard et al., 2005). Additionally, the presence of APOE4 enhances the attachment of Cp elementary bodies to human cells (Gérard et al., 2008). Another study observed that Cp infection was associated with worse memory among APOE ε4 carriers, but not among non–ε4 carriers (Zhao et al., 2020). APOE4 has been shown to increase the accumulation of lipid droplets in microglial cells and negatively affect cholesterol efflux (Leeuw et al., 2022; Victor et al., 2022). Cp requires host-derived lipids for growth and limits cholesterol efflux in macrophages (Ooij et al., 2000; Tumurkhuu et al., 2018). As APOE4 is highly expressed in astrocytes, this may allow Cp to grow more readily in the astrocytes of APOE4 carriers (Tcw et al., 2022). Thus, the presence of APOE4 may facilitate enhanced Cp growth beyond the increased attachment to cells observed *in vitro*.

### SIRT 1

Sirtuin 1 is known to be beneficial to multiple age-related neurological disorders including AD. SIRT1 plays a critical role at the nexus between many cellular pathways including metabolism, energy production, and stress responses (Liu et al., 2008). Low levels of SIRT1 are associated with accumulation of Aβ and tau in the brain cerebral cortex of AD patients and SIRT1 has become a target for therapeutic efforts (Julien et al., 2009; Liu et al., 2022). Upon infection by Cp, the SIRT1 pathway is reduced in macrophages (Taavitsainen-Wahlroos et al., 2022). Resveratrol, the potent SIRT1 inducer, inhibits the growth of the closely related organism, *Chlamydia trachomatis* (Petyaev et al., 2017). Levels of SIRT1 is reported to be downregulated in AD conditions. Chen et al. observed that SIRT1 protects against microglia-dependent Aβ toxicity by inhibiting NF-κB signaling (Chen et al., 2005). Supportively, another group suggested that SIRT1 activation in neurons controls calorie restriction which can prevent amyloid neuropathy and AD (Qin et al., 2006). Kim et al. also found a neuroprotective effect of SIRT1 against AD (Kim et al., 2007). Low levels of SIRT1 was associated with a higher amount of accumulated tau in AD (Julien et al., 2009). SIRT1 deletion resulted in increased acetylated-tau phosphorylated-tau, possibly by blocking proteolysis (Min et al., 2010). SIRT1 inhibition is also reported to be associated with a higher bacterial load during infection (Hajra et al., 2023). Additionally, SIRT1 activation leads to increased cholesterol efflux via upregulation of ABCA1 (Feng et al., 2018). Overall, the sirtuin-1 pathway appears to act against both infectious processes as well as AD pathogenesis and its downregulation may be a key step in infection exacerbated AD.

### MMP-9

MMP-9 is a critical pathogenic mediator in several CNS disorders, including AD (Gu et al., 2020). MMP9 is associated with Aβ transport and abnormal tau cleavages thus facilitating the brain Aβ accumulation and tau oligomerization (Nübling et al., 2012; Shackleton et al., 2019; Hernandes-Alejandro et al., 2020). The plasma level of MMP-9 is increased in AD patients, and its expression is elevated in postmortem AD brain tissues (Lorenzl et al., 2003). MMP-9 plays an important role in *C. trachomatis-*induced trachoma (Natividad et al., 2006). Cp Heat Shock Protein 60 regulates the expression of MMP-9 expression in macrophages (Kol et al., 1998). Microglial cells infected with Cp express MMP-9 and TNF-α which can further trigger the production of inflammatory cytokines (Ikejima et al., 2006). Upregulation of extracellular MMP inducers and gelatinases were reported in human atherosclerotic patients infected with Cp (Choi et al., 2002) and MMP-9 expression is associated with the presence of Cp in human coronary atherosclerotic plaques (Arno et al., 2005). Collectively, Cp infection induced the production of MMP-9 which subsequently triggered inflammatory cascades in AD.

### NLRP3 inflammasome

The nucleotide binding domain and leucine-rich repeat (NLR) pyrin domain containing three (NLRP3) inflammasome is a major driver of diverse inflammatory cascades in CNS diseases, including AD (Shao et al., 2018; Jha et al., 2023). Activation of the NLRP3 inflammasome generally requires two distinct steps: (1) NF-κB signaling to upregulate inflammasome components, and (2) activation and formation of the inflammasome via danger signals (Evavold and Kagan, 2019). The formation of the NLRP3 inflammasome leads to caspase-1 activation which can then cleave pro-IL-1β and pro-IL-18 into their active mature forms and subsequently secreted. In AD, NLRP3 inflammasome signaling is triggered by multiple factors such as Aβ, tau, activated glial cells, impaired autophagy, and endoplasmic reticulum stress that ultimately leads to the release of pro-inflammatory cytokines such as IL-1β and IL-18 (Jha et al., 2023). These inflammatory molecules are associated with neuroinflammation, neurodegeneration, and cognitive deficit in AD (Liang et al., 2022). Most pathogens promote NLRP3 inflammasome activation by increasing NF-κB downstream signaling and phagolysosome impairment and or mitochondrial dysfunction (Anand et al., 2011). Cp can directly activate the NLRP3/ASC inflammasome in Cp-infected bone marrow-derived macrophages, leading to the release of biologically active IL-1β (He et al., 2010; Itoh et al., 2014) Caspase-1 and IL-1β are required to control acute pulmonary Cp infection in mice, and modulation of inflammasome activity via autophagy can lead to increased pathology (Shimada et al., 2011; Crother et al., 2019). While there is lack of studies examining the role of Cp in NLRP3 inflammasome signaling in AD, Cp triggering inflammasome activation in other disease/cell types indicate that Cp infection may promote NLRP3 mediated inflammatory responses in AD as well.

### Other biomolecules

In addition to those listed above, there are several other biomolecules which may provide a link between Cp infection and AD pathologies. These include: TREM 2 (N'Diaye et al., 2009; Gratuze et al., 2018), Lipoprotein receptor-related protein 1 (Liu et al., 2017; Al-Atrache et al., 2019), Homocysteine inducible ER protein with ubiquitin like domain (Li et al., 2021; Wen et al., 2021), inducible nitric oxide synthases (Igietseme et al., 1998; Shao et al., 2010; Kummer et al., 2011), MAP1B (Al-Younes et al., 2011; Ma et al., 2014), Midkine (Muramatsu, 2011; Sanino et al., 2020), aldolase A (Ende and Derré, 2020; Li et al., 2021), gamma-actin 1 (Bagnicka et al., 2021; Li et al., 2021), DDX5 (Li et al., 2021; Hu et al., 2022), Osteopontin (Filippis et al., 2017; Rentsendorj et al., 2018; Kessler et al., 2019), and ICAM-1 (Igietseme et al., 1999; Vielma et al., 2003). However, their direct relationship with Cp infection in AD is yet to be established.

## Conclusion

Accumulation of abnormal Aβ and tau forms, alongside neurodegeneration are the defining events making up AD pathogenesis (Jack et al., 2016, 2018). These events are further underpinned by other adjunctive ones, such as inflammation, vascular dysfunction, synaptic loss, and functional impairments. The exact cause of late-onset AD is still not understood but it is likely multifactorial. The concept that infections are a key element in AD has gained support recently. The data presented here in this review article provides strong correlative support for Cp playing a contributory role in AD. However, further investigations need to be performed in both human and animal studies to understand what role Cp infection might actually play and by what mechanism Cp induces or exacerbates AD. The optimization of experimental protocols and methods of testing will also help to better understand and confirm the presence of Cp in the AD brain. In addition to the standardized retrospective studies, we should also perform multi-center studies with increased cohorts. Controlled animal studies using AD transgenic models with Cp infection will provide valuable insight into this question. In addition to Cp infection, several other studies confirmed the presence of different microbes in AD and other dementia types, hence multi-pathogen studies including both viruses and bacteria should also be taken into consideration in future studies. The gap in finding appropriate therapeutic approaches against AD can be resolved with the utilization of specific therapy or preventative measures against the responsible microbes in AD pathogenesis. Overall, among the various microbes that have been studied to have a role in AD, Cp appears to be the most plausible agent therefore warranting further studies.

## Author contributions

LS: Conceptualization, Writing – original draft, Writing – review & editing. BG: Conceptualization, Writing – review & editing. YK: Conceptualization, Writing – review & editing. MK-H: Conceptualization, Writing – original draft, Writing – review & editing. TC: Writing – original draft, Writing – review & editing.
